# Clinical Characteristics, *In Silico* Analysis, and Intervention of Neonatal-Onset Inflammatory Bowel Disease With Combined Immunodeficiency Caused by Novel *TTC7A* Variants

**DOI:** 10.3389/fgene.2022.921808

**Published:** 2022-06-16

**Authors:** Yun-e Chen, Jingfang Chen, Wenxing Guo, Yanhong Zhang, Jialing Li, Hui Xie, Tong Shen, Yunsheng Ge, Yanru Huang, Wenying Zheng, Mei Lu

**Affiliations:** ^1^ Department of Pediatrics, Women and Children’s Hospital, School of Medicine, Xiamen University, Xiamen, China; ^2^ Department of Gastroenterology, Xiamen Branch of the Children’s Hospital of Fudan University (Xiamen Children’s Hospital), Xiamen, China; ^3^ Department of Ultrasound Medicine, Women and Children’s Hospital, School of Medicine, Xiamen University, Xiamen, China; ^4^ Prenatal Diagnostic Center Laboratory, Women and Children’s Hospital, School of Medicine, Xiamen University, Xiamen, China; ^5^ Genokon Institute of Medical Science and Laboratory, Xiamen, China

**Keywords:** inflammatory bowel disease, immunodeficiency, neonatal, *TTC7A*, intervention

## Abstract

We aimed to explore the genotypic and phenotypic characteristics of neonatal-onset inflammatory bowel disease (IBD) with combined immunodeficiency due to *TTC7A* mutation. We examined the clinical manifestations, imaging results, endoscopic and histological findings, interventions, and prognosis of a proband with neonatal-onset IBD and performed biochemical analyses, whole-exome sequencing (WES), and *in silico* analysis. Our proband developed severe early-onset diarrhea, malnutrition, electrolyte imbalance, dehydration, and recurrent infections after birth. Radiographic and ultrasonic images showed no specific manifestations. Endoscopic and histological examination revealed chronic inflammation. Immune function examination indicated immunodeficiency. WES identified compound heterozygous *TTC7A* mutations (c.2355+4A>G, c.643G>T) in the proband. In the expression analysis, no abnormal splicing in the *TTC7A* sequence was observed due to the c.2355+4A>G mutation; however, the mRNA expression was reduced. The proband’s condition did not improve after treatment with methylprednisolone or leflunomide. The proband died when treatment was stopped at the age of 5 months and 19 days. Compound heterozygous mutations (c.2355+4A>G, c.643G>T) in the *TTC7A* gene are described and verified for the first time. Our report expands the phenotypic spectrum of *TTC7A* mutations and the genotypic spectrum of very early-onset IBD with combined immunodeficiency.

## Introduction

Very early onset inflammatory bowel disease (VEOIBD) encompasses a group of diseases in children aged less than 6 years (Kelsen et al., 2019); it has a unique clinical presentation with severe colonic disease in infancy (Griffiths, 2004; Muise et al., 2012a). Mutations in *IL10RA/B*, *IL106*, *XIAP7*, *ADAM178*, *NCF49*, *NCF2/RAC210*, and *TTC7A* cause a severe form of VEOIBD, with symptoms developing in infancy (Glocker et al., 2009; Matute et al., 2009; Glocker et al., 2010; Blaydon et al., 2011; Worthey et al., 2011; Muise et al., 2012b; Kelsen and Sullivan, 2017). *TTC7A*, a member of the tetratricopeptide repeat (TPR) domain-containing proteins, has multiple functions in cell cycle control, phosphate turnover, and protein transport and secretion (White et al., 2005). *TTC7A* gene mutations occur in several patients with multiple intestinal atresia (MIA) and gastrointestinal defects, immunodeficiency syndrome, and inflammatory bowel disease (IBD) with or without immunodeficiency; they are inherited in an autosomal recessive manner (OMIM: 609332). Approximately 50 cases of TTC7A deficiency have been reported worldwide, most of which are MIA with combined immunodeficiency (CID), and only a few are IBD cases (Jardine et al., 2019a). Missense *TTC7A* mutations often result in clinical features of VEOIBD, and truncating *TTC7A* mutations (nonsense, frameshifts, or large deletions) are associated with greater morbidity and mortality in MIA-CID patients (Samuels et al., 2013; Avitzur et al., 2014; Bigorgne et al., 2014). Patients with mutations affecting the splice acceptor sites present with intestinal atresia, increased intestinal cell apoptosis, and severe bowel inflammation (Samuels et al., 2013); however, it is difficult to establish a significant correlation between the genotype and phenotype. Most patients were diagnosed within 1 year after birth; only two patients were diagnosed in the neonatal period (Jardine et al., 2019a). Neonatal-onset IBD often exhibits a poor response to conventional treatments in contrast to adult-onset IBD (Heyman et al., 2005).

In this study, we describe the clinical presentation, imaging results, pathological features, functional analysis, treatment, and prognosis of a proband with neonatal-onset IBD caused by two novel *TTC7A* compound heterozygous mutations. There was no improvement in the proband’s condition after treatment with methylprednisolone or leflunomide. This is the first known case of *TTC7A* mutations in mainland China and it extends the pathogenic spectrum of this gene.

## Materials and Methods

### Proband

We retrospectively studied a proband from the Women and Children’s Hospital, School of Medicine, Xiamen University, China. All relevant clinical information was collected, including clinical progression, laboratory findings, imaging, and endoscopic and histological characteristics. Stool electrolytes were detected using biochemical testing methods.

### Whole-Exome Sequencing and Variant Classification

Peripheral blood samples of the proband, his parents, and control (a normal peer) were collected, and trio-based WES was performed at Guangzhou KingMed Diagnostics Group Co. Ltd. (Guangzhou, China). Genomic DNA was extracted using the QIAamp DNA Mini Kit (Qiagen, Hilden, Germany), and mechanical shearing was performed using Covaris AFA-energetics^®^ (Covaris Inc., Woburn, MA, United States) according to the manufacturer’s instructions. DNA library preparation and adaptor hybridization were performed according to the standard procedures of the Agencourt AMPure XP Beads (Beckman Coulter Inc., Brea, CA, United States). The DNA library was used to capture and collect DNA from the target exons and adjacent splice sites, using the Agilent SureSelect Human All Exon V6 kit (58 M). Finally, PE150 + 150 sequencing was performed using Illumina NovaSeq 6000 platform (Illumina, Inc., San Diego, CA, United States). The reads were mapped to the reference human genome (UCSC hg19).

Primers were designed to amplify the candidate regions of the mutations identified using WES. Subsequently, Sanger sequencing using ABI PRISM 3130XL gene analyzer (Applied Biosystems, California, United States) was performed.

### *In Silico* Analysis

All variants were compared with the data in the 1000 Genomes Project (https://www.internationalgenome.org), Exome Aggregation Consortium (ExAC) (https://exac.broadinstitute.org/), and dbSNP (https://www.ncbi.nlm.nih.gov/snp/) databases. Variants with greater than 0.01 minor allele frequency in the control databases were excluded. Pathogenicity predictions for proteins were performed using Revel, SIFT, PolyPhen2, PROVEAN_pred, LRT_pred, Mutation Taster, etc.

### Analysis of *TTC7A* Expression

Total RNA was extracted from the peripheral blood lymphocyte cell lines using PowerUpTM SYBR Green Master Mix Kit (Life Technologies). The mRNA was reverse transcribed to cDNA using RevertAid First Strand cDNA Synthesis Kit (Thermo Scientific). The region near the splice site was amplified using the primers, 5′-TCC​CCA​CTT​CTC​ACT​CAG​TAC​TC-3′ and 5′-GTG​GCA​CGT​ACT​CTG​CCT​CT-3′ (product length: 218 bp), followed by agarose gel electrophoresis and sequencing through Sanger sequencing. Real-time quantitative PCR (qPCR) was performed to verify whether the splice site mutation affected the mRNA expression. The relative standard curve method was used to analyze the expression level. The expression was normalized to that of*ACTB* in the same sample; three biological repeats were measured. The real-time PCR primers used were: *TTC7A*, 5′-ATG​CAT​AGC​CTG​GGT​CTG​AT-3′ and 5′-GTG​GCA​CGT​ACT​CTG​CCT​CT-3′ (product length: 96 bp); *ACTB*, 5′-TGG​TGC​CAG​ATT​TTC​TCC​A-3′ and 5′-GGC​ATG​GGT​CAG​AAG​GAT​T-3′ (product length: 128 bp).

### Informed Consent

Written informed consent for genetic testing and the publication of research data was obtained from the proband’s parents. The protocol was approved by the Women and Children’s Hospital Ethics Committee (KY-2020-011).

## Results

### Clinical Features of the Proband

The proband, a male infant conceived *via in vitro* fertilization, was born to non-consanguineous Chinese parents at 35 weeks and 6 days of gestation. The clinical characteristics of his parents were normal; and the mother and father were aged 27 and 29 years, respectively. No complications were observed during the prenatal period. No exposure to toxic or harmful substances during pregnancy was reported. There was no family history of genetic diseases. His birth weight and length were 2,800 g and 50 cm, respectively. He was breastfed after birth and presented with diarrhea with yellow, watery, and bloody stool 5–10 times per day. He was admitted to a tertiary children’s hospital; however, he showed no improvement after 1 week of antimicrobial treatment. He was admitted to our hospital for further treatment, at the age of 1 month and 8 days, with a weight of 2,600 g, head circumference of 33 cm, and body length of 50 cm, without dysmorphic features. The proband had malnutrition, moderate dehydration, and severe perianal dermatitis.

Because of the large quantity of watery stool (approximately 300–940 ml/day), total parenteral nutrition, amino acid milk powder, deep hydrolyzed protein milk powder, or breast milk were administered after admission; however, there was no improvement in his condition. The stool was considered to be high-output secretory diarrhea. Severe hypokalemia, hyponatremia, and metabolic acidosis occurred, and the proband recovered after total parenteral nutrition every day. The erythrocyte sedimentation rate was normal; however, the ferritin levels were >1,500 ng/ml (reference value <200 ng/ml). Interleukin (IL)-6 levels reached 74.6 pg/ml (reference value ≤5.9 pg/ml), and the tumor necrosis factor-α level was 39.2 pg/ml (reference value ≤ 8.1 pg/ml). A diagnosis of IBD was suspected.

During hospitalization, the proband had recurrent fever; blood immune function investigation indicated CID (Table 1). The test for cytomegalovirus antibody in the blood was negative; however, the amounts of cytomegalovirus DNA in the blood and urine were 4.12 × 10^6^ copies/ml and 1.47 × 10^6^ copies/ml, respectively. Cytomegalovirus and *Pneumocystis carinii* were detected in the bronchoalveolar lavage fluid through metagenomic detection; *Candida albicans* was detected in urine culture and sputum culture. The risk factors for these infections include malnutrition, CID, long hospital stays, and long-term use of antibiotics and hormones.

**TABLE 1:** Lymphocyte subsets and immunoglobulin levels of the proband.

	Day 52^a^	Day 65	Day 78	Day 105^b^	References
IgA (g/L)	0.35	<0.28	<0.28	0.06	0.05–0.60
IgG (g/L)	5.51	3.93	1.4	5.1	2.75–7.50
IgM (g/L)	0.26	0.53	0.34	0.29	0.10–0.70
C3 (g/L)	0.65	0.98	1.04	0.55	0.70-1.40
C4 (g/L)	0.15	0.15	0.18	0.14	0.10–0.40
CD3^+^T (10^9^/L)	0.77	—	—	2.04	1.85–4.02
CD3^+^CD4^+^T (10^9^/L)	0.56	—	—	0.32	1.33–3.11
CD3^+^CD8^+^T (10^9^/L)	0.21	—	—	1.45	0.66–1.15
NK (10^9^/L)	0.07	—	—	5.16	0.27–0.73
B (10^9^/L)	0.50	—	—	16.93	0.34–2.09

a 2 days after 5 g IgG infusion.

b 3 days after 5 g IgG infusion.

The proband received cefotaxime, piperacillin-tazobactam, cefoperazone-sulbactam, meropenem, ganciclovir, co-trimoxazole, fluconazole, voriconazole, and intravenous immunoglobulin (IVIG). Methylprednisolone 1–2 mg/kg/day was given intravenously for 11 days. He received leflunomide (the dose increased over time from 0.5 to 1.0 mg/days) for 4 weeks, commencing at the age of 4 months 23 days. Although his infections were under control after comprehensive treatment, he still presented with refractory diarrhea, and his weight remained in the range of 2,300–3,400 g after 4 months. The infant died when his parents discontinued treatment at the age of 5 months and 19 days.

### Imaging Features and Endoscopic and Histological Characteristics

**FIGURE 1:**
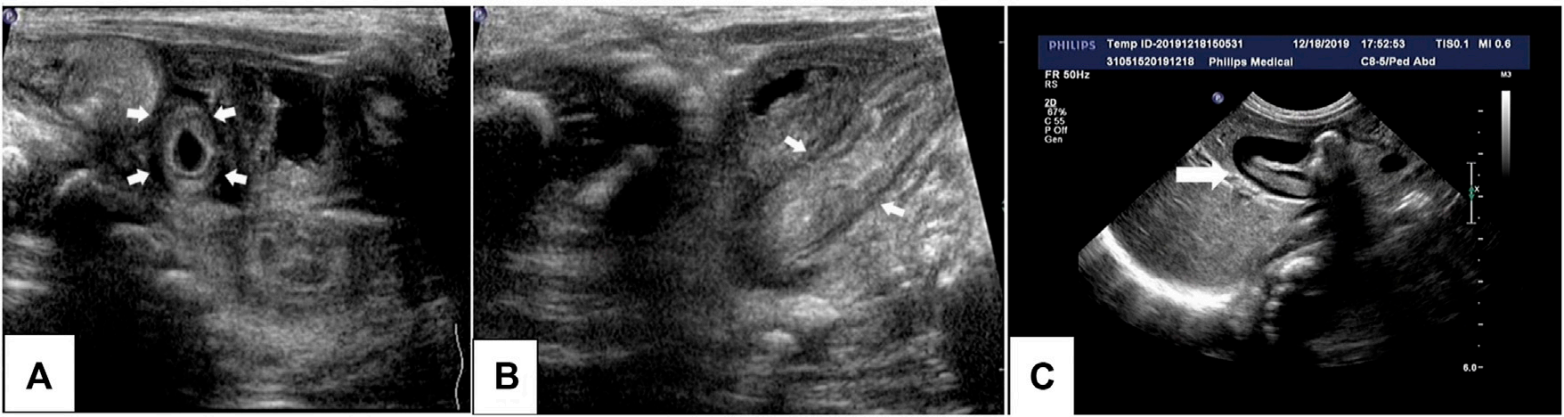
Ultrasonography of the proband’s abdomen. Thickening of the small intestine and colon, enhanced echogenicity of the mucous membrane and submucosa, and effusion in the colon are visible **(A,B)**. Low-echogenicity area in the gallbladder (bile sludge) **(C)** can be seen, which disappeared the next day.

**FIGURE 2:**
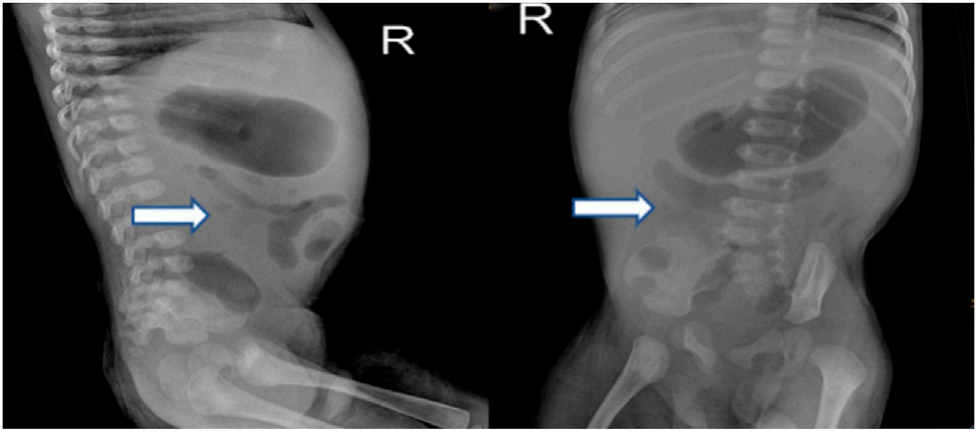
X-ray of the proband’s abdomen revealed stiffness in the intestines and low volume of intestinal gas.

**FIGURE 3:**
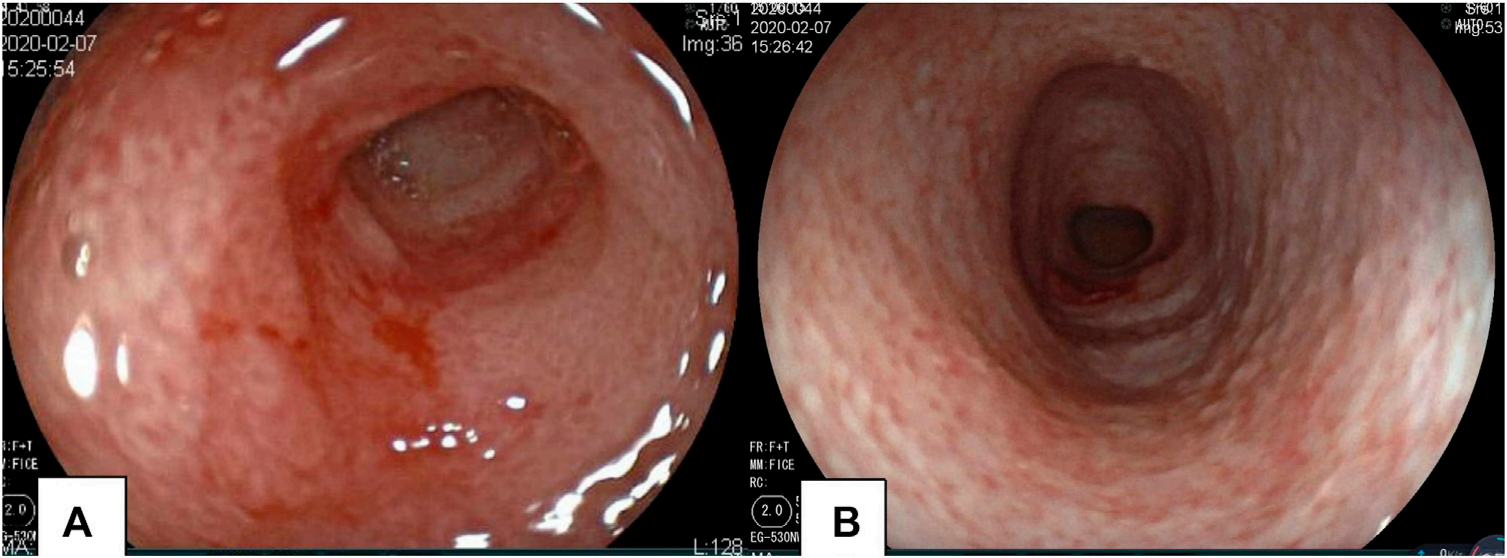
Gastroscopy and colonoscopy of the proband. Mucosal erosion and edema of the pylorus revealed by gastroscopy **(A)**. Colonoscopy revealed the mucosa of the whole colon to be rough; the surface was scattered with a white pseudomembrane. Part of the intestinal mucosa was eroded, and the intestine was slightly narrow **(B)**.

**FIGURE 4:**
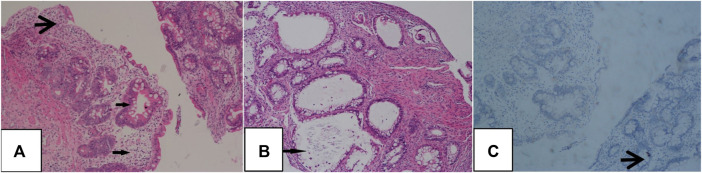
Histopathological findings of the proband’s intestine. Chronic mild superficial inflammatory changes are seen in the esophagus and stomach. The duodenal crypt was atrophied, and plasma cells were scattered in the interstitium **(A)**. Twisted colon crypts, mucus hypersecretion **(B)**, focal epithelial hyperplasia, and basal plasmacytosis are evident in the large intestinal mucosa **(C)**.

The proband underwent abdominal ultrasound, radiography, gastroscopy, colonoscopy, and biopsy pathological analyses (Figures 1–4). Gastroscopy and colonoscopy examination were performed on days 98 and 100, respectively.

### WES and Variant Classification

**FIGURE 5:**
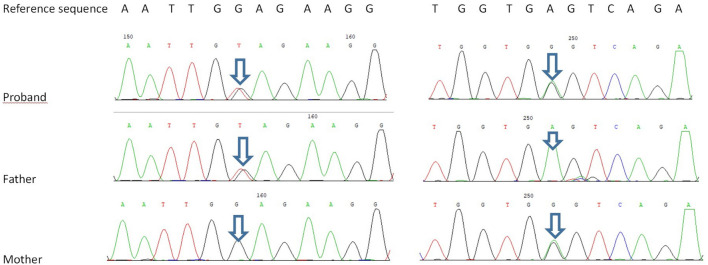
Sanger sequencing validation of the *TTC7A* variants.

Trio-based WES identified two novel heterozygous variants in *TTC7A*: a mutation in exon 4, c.643G>T, inherited from his father, and another in exon 19, c.2355+4A>G inherited from his mother. According to the American College of Medical Genetics and Genomics and the Association for Molecular Pathology variant-interpretation guidelines (Richards et al., 2015) and the work groups from ClinGen (https://clinicalgenome.org/), the nonsense mutation c. 643G>T causes premature termination of the protein at amino acid 215 (p. Glu215X); the variant is classified as a pathogenic variant (PVS1 + PM2-supporting + PP3). The intronic allele c.2355+4A>G is predicted to affect mRNA splicing of TTC7A transcripts, according to dbscSNV_ADA and dbscSNV_RF; the variant is classified as a variable of uncertain significance (PM2-supporting + PP3; Figure 5).

### *TTC7A* Expression Analysis

**FIGURE 6:**
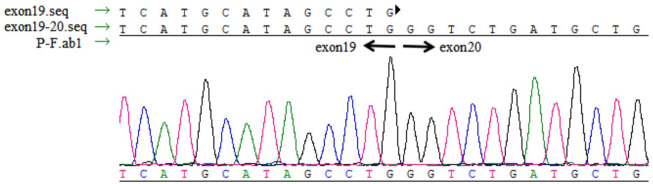
Complementary DNA sequencing of the proband.

**FIGURE 7:**
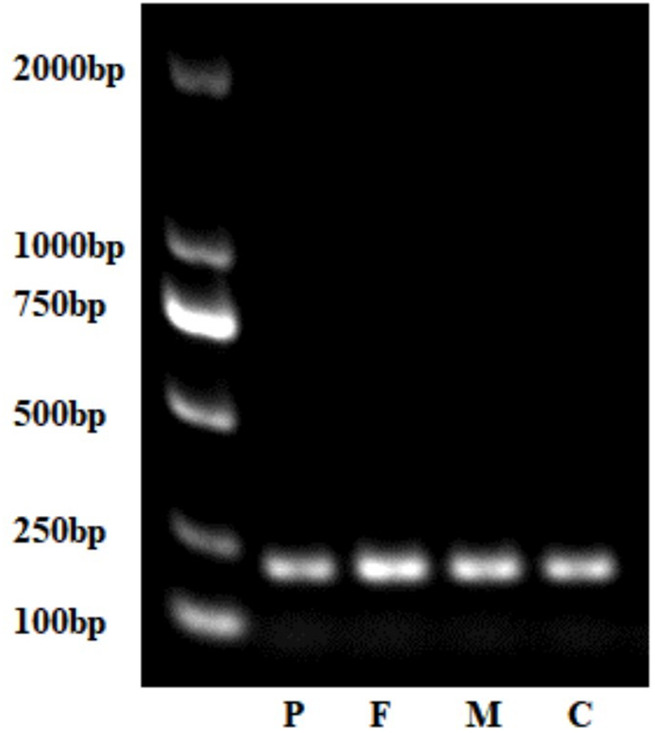
Gel electrophoresis of complementary DNA of the proband, parents, and control (normal peer). P, proband; F, father; M, mother; C, control.

**FIGURE 8:**
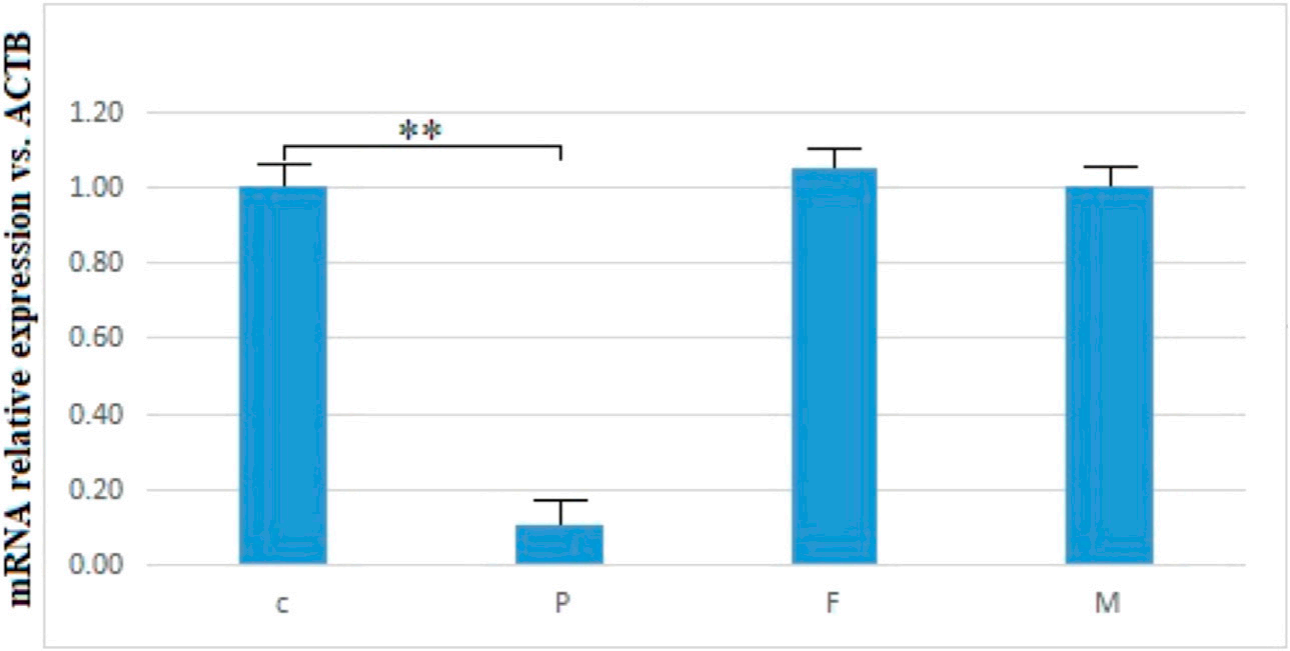
mRNA relative expression of *TTC7A* vs. *ACTB* of the proband, parents, and control. **p < 0.01. P, proband; F, father; M, mother; C, control.

No abnormal splicing bands were observed in the agarose gel electrophoresis; no abnormal splicing sequences were detected using Sanger sequencing. However, the expression of *TTC7A* mRNA decreased in the proband (*p* < 0.01), which could have resulted in a decrease in the protein levels. However, no aberrant expression of TTC7A was detected in the parents (Figures 6–8). The decrease in mRNA expression could lead to decreased protein expression or the production of the wrong protein (Chen et al., 2013; Bigorgne et al., 2014).

## Discussion

The global incidence rate of IBD is 37.5/100,000 annually. In the United States, the incidence of IBD was the highest in adults (18–59 years) and older adults (>60 years) from 2005 to 2015 and the lowest in pediatric patients (0–17 years) (Keyashian et al., 2019). In a study of 1,412 pediatric cases, VEOIBD accounted for approximately 3% of cases, and infantile-onset IBD accounted for only 1%. The incidence of VEOIBD remained stable over the study period (1988–2011); however, the incidence of EOIBD increased (Bequet et al., 2017). These results suggest a probable genetic origin for VEOIBD, whereas the increased incidence in older children may be linked to environmental factors (Bequet et al., 2017). The most important clinical sign of monogenic IBD is the young age at onset. In this study, the case was initially thought to be related to genetic factors because of the very early onset and severe symptoms.

Neonatal or infantile-onset IBD is often associated with primary immune deficiency disorders, especially those caused by mutations in *IL-10*, *XIAP*, *NCF2*, *IPEX*, and *TTC7A* (Kelsen and Sullivan, 2017). *TTC7A* has 20 exons and nine TPR domains, and mutations in exons 2, 7, and 20 are relatively common (Lien et al., 2017). TPR domains are structurally conserved helix-turn-helix motifs that play an important role in protein scaffolding and have large surface areas that accommodate multiple protein interactions (Blatch and Lässle, 1999; Zeytuni and Zarivach, 2012). No case of *TTC7A* mutation was reported in mainland China, before this case. Our proband had two novel *TTC7A* mutations, c.643G>T in exon 4, inherited from his father, and c.2355+4A>G in exon 19, inherited from his mother, which was not described in the Human Gene Mutation Database. *TTC7A* mutation exhibits an autosomal recessive inheritance pattern; the proband’s parents have normal phenotypes. The c.643G>T mutation, a nonsense mutation in exon 9, results in premature termination of the protein and is predicted to cause loss of function. qPCR showed that the c.2355+4A>G mutation decreased *TTC7A* mRNA expression. We believed that this intron mutation would affect splicing; however, the experimental results revealed that this was not the case.

To date, few studies have performed functional analysis of the specific variants of *TTC7A*. We did not perform immunostaining analysis for assessing the proteins; however, we speculate that the c.2355+4A>G mutation could affect the TPR domain, which binds and recruits phosphatidylinositol 4 (PI4)-kinase III alpha to the plasma membrane. This process facilitates the synthesis of mutant PI4-phosphate affecting the TPR domain, resulting in negative phenotypes and poor outcomes (Lien et al., 2017). Studies on truncating *TTC7A* mutations suggested that nonsense-mediated decay of *TTC7A* mRNA transcripts showed an obvious loss of protein, consistent with the increased phenotypic severity in patients with MIA--CID, whereas non-truncating mutations tended to present as VEOIBD (Samuels et al., 2013; Avitzur et al., 2014). Nonsense mutations cause severe intestinal diseases (e.g., MIA) and CID; however, missense mutations mainly present as IBD and mild intestinal disease (Lawless et al., 2017; Neves et al., 2018). Our proband had a truncated mutation that affected the TPR domain, which manifested as neonatal-onset IBD and CID, but not as MIA. It is difficult to attribute complex phenotypes to physical alterations in TTC7A.

Fewer than 10% of patients with *TTC7A* mutations demonstrate VEOIBD with secretory diarrhea, chronic intestinal inflammation, lymphocytopenia, and/or hypoglobulinemia (Lien et al., 2017; Jardine et al., 2019a). Patients with VEOIBD are more likely to present with ileal involvement and isolated colonic involvement. Rectal bleeding and mucous stools with weight loss and underlying primary immunodeficiency appear to be more common in VEOIBD cases with monogenic causes (Bequet et al., 2017; Kelsen et al., 2019; Shim, 2019).

Radiography and ultrasonography are the most commonly used methods of examination in pediatric patients, especially before the diagnosis of IBD. No specificity or significant differences between pediatric and adult IBD patients were identified (Horsthuis et al., 2008). Imaging studies of VEOIBD are relatively rare. Hepatosplenomegaly, significant intestinal wall thickening, and mildly dilated bowels are reported in neonatal IBD (Barber et al., 2018). In our proband, abdominal radiography revealed stiffness of the intestines and decreased intestinal gas volume. Gastrointestinal ultrasonography revealed thickening of the bowel wall, reduction in intestinal gas volume, and fluid accumulation in the colon. All these imaging findings were consistent with the pathologic changes of apoptosis, twisted crypts, and hypersecretion of mucus in the colon. It is very difficult to perform gastroenteroscopy (GE) in the early neonatal or infant stages because of the severe risk conditions, poor bowel preparation, and technical failure. The proband underwent GE at approximately 3 months of age, and chronic mild superficial inflammation in the esophagus, stomach, and duodenum, twisted crypts, and hypersecretion of mucus in the colon were observed. These features, unlike that in the atypical tissues observed in older children and adult ulcerative colitis or Crohn’s disease, mainly involve the colon. Compared to that in adult IBD patients, extensive ileocolonic inflammation is more predominant in pediatric IBD patients (Peloquin et al., 2016). The most common endoscopic findings are mucosal bleeding among patients with VEOIBD and visual ileitis or ileocolonic bleeding in older patients with IBD (Conrad et al., 2019).

Severe chronic structural changes, increased frequency of apoptosis, blunt villi in the small intestine, and eosinophils in the surface epithelium, crypts, and lamina propria reported in VEOIBD are discovered in some primary immunodeficiencies (Conrad et al., 2019). T-cell maturation disruption and lymphocytopenia owing to a hypoplastic thymus and low blood immunoglobulin levels caused by increases in the relative levels of transitional B cells and B-cell receptor assembly and organelle synthesis in activated B cells are speculated (Avitzur et al., 2014; Woutsas et al., 2015). Therefore, immunodeficiency is closely related to VEOIBD; immune function should be tested as soon as possible during diagnosis. According to the data from our case, the phenotype involves extensive lesions in the stomach, duodenum, small intestine, and colon, leading to very early onset, severe symptoms, and immunodeficiency.

Most monogenic IBD patients are resistant to conventional medical treatment (Heyman et al., 2005; Kelsen et al., 2019; Shim, 2019), as are patients with neonatal-onset IBD with *TTC7A* deficiency. Currently, treatment options include early intestinal resection (in MIA), parenteral nutrition, and regular immunoglobulin infusion (Kammermeier et al., 2016). Total or partial parenteral nutrition has been reported in many patients with *TTC7A* mutations presenting with MIA, gastrointestinal defects, and IBD (Avitzur et al., 2014; Bigorgne et al., 2014; Lemoine et al., 2014); early application of parenteral nutrition is necessary because of intestinal malabsorption and excessive intestinal loss. In our study, parenteral nutritional support was initiated early.

VEOIBD shows poor response to immunosuppressives, steroids, and biologics (Jardine et al., 2019a). Hematopoietic stem cell transplantation treatment has been reported for monogenic IBD, which may restore immunity and increase survival in immunodeficient patients; however, it does not appear to improve the phenotypes associated with bowel epithelial defects (Jardine et al., 2019a). The survival rate of patients with *TTC7A* deficiency treated with HSCT did not improve (Kammermeier et al., 2016; Fayard et al., 2018). Intestinal and liver transplantation restores intestinal and immune functions in a child with MIA-CID (Gilroy et al., 2004). Unfortunately, intestinal transplantation is not feasible because of the limitations of donor factors. *In vitro* studies show that Rho A inhibitors (Y-27632) and leflunomide are effective treatments (Notarangelo, 2014; Jardine et al., 2019b); however, no *TTC7A*-deficient patients have received leflunomide. After we obtained consent from the parents, we started leflunomide treatment for 4 weeks but without any success. The efficacy of leflunomide *in vivo* is uncertain. Leflunomide is mainly used for treating rheumatoid arthritis. Leflunomide was effective in an *in vivo* zebrafish study on TTC7A deficiency; however, this drug was not effective in our proband. Further elucidation of the pathogenesis and therapeutic targets in TTC7A-deficiency through the accumulation of clinical experience is warranted. The infant died when treatment was stopped at the age of 5 months and 19 days.

The *TTC7A*-deficient genotype may be closely related to prognosis. The average survival of patients with *TTC7A* nonsense mutations is less than 12 months. Some patients with biallelic missense mutations that do not result in autoimmune disorders or involve TPR domains have a relatively better prognosis and can survive to adulthood (Lawless et al., 2017; Neves et al., 2018).

## Conclusion

Two novel compound heterozygous mutations in *TTC7A* were identified for the first time in mainland China. These mutations manifested as neonatal-onset IBD-CID with a poor prognosis. No specific manifestations were found with imaging or endoscopy. Studying the genotype and clinical phenotype of TTC7A deficiency requires the accumulation of more clinical data and in-depth functional research.

## Data Availability

The datasets for this article are not publicly available due to concerns regarding participant/patient anonymity. Requests to access the datasets should be directed to the corresponding authors.
